# Circulating MicroRNAs as Prognostic Molecular Biomarkers in Human Head and Neck Cancer: A Systematic Review and Meta-Analysis

**DOI:** 10.1155/2019/8632018

**Published:** 2019-11-18

**Authors:** Shree Ram Lamichhane, Thanuja Thachil, Harriet Gee, Natalie Milic

**Affiliations:** ^1^College of Human and Health Sciences, Charles Darwin University, Darwin, NT, Australia; ^2^Alan Walker Cancer Care Centre, Northern Territory Radiation Oncology, Darwin, NT, Australia; ^3^Central Coast Cancer Centre, Gosford Hospital, NSW, Australia; ^4^Department of Radiation Oncology, Crown Princess Mary Cancer Centre, Westmead Hospital and School of Medicine, University of Sydney, Australia

## Abstract

**Background:**

Circulating microRNAs (miRNAs) are potential molecular biomarkers for cancer detection; however, little is known about their prognostic role in head and neck cancer. This current study is aimed at evaluating the role of novel miRNAs in the survival of head and neck cancer patients.

**Materials and Methods:**

We performed a systematic literature search using online databases for articles published between December 2006 and February 2019. A meta-analysis was conducted to assess the correlation between miRNA expressions and overall survival (OS) among the selected head and neck cancer studies. After multilevel screening by reviewers, meta-analysis was performed using hazard ratios (HR) and associated 95% confidence interval (CI) of survival to calculate a pooled effect size.

**Result:**

A total of 1577 patients across 13 studies were included in the literature review, with 18 miRNAs upregulated and 4 miRNAs downregulated predicting a poor overall survival. The forest plot generated using cumulated survival data resulted in a pooled HR value of 2.943 (95% CI: 2.394-3.618) indicating a strong association of dysregulated miRNA expression with a poor outcome. Only 2 miRNAs—low levels of miR-9 and high levels of miR-483-5p—were observed in two studies, both showing a significant association with overall cancer survival.

**Conclusion:**

To our knowledge, this is the first comprehensive systematic review and meta-analysis that examines the prognostic role of circulating miRNAs from blood in head and neck cancer patients. The combined effect estimates a HR across multiple studies and also supports the previous individual findings that an alteration in miRNA expression is highly associated with poor prognosis. This has the potential to use serum and/or plasma miRNAs as biomarkers and become novel tools for predicting the prognosis of head and neck cancer patients in the near future.

## 1. Introduction

Head and neck cancers are a group of epithelial malignancies that occurs inside the sinuses, nose, mouth, and salivary glands including the throat. According to the global cancer statistics (2018), the annual incidence of the head and neck cancers worldwide is more than 650000 cases with approximately 300000 deaths each year. It is also estimated that males are affected significantly more than females, with a ratio ranging from 2 : 1 to 4 : 1 [1, 2]. Although advanced surgical techniques and effective adjunctive therapeutics have been developed, the overall survival of patients is still poor due to its heterogeneous nature, late diagnosis and development of local recurrences, distant metastases, and second primary tumors [3, 4]. Blood-based prognostic biomarkers are particularly attractive as they are minimally invasive, but there are currently none in routine clinical use; hence, detection of accurate blood biomarkers could help to prognosticate and tailor treatment in order to improve outcomes and minimise treatment-related morbidity and mortality.

miRNAs are a family of short (18-25 nucleotides), evolutionarily conserved, single-stranded, noncoding RNA molecules that regulate numerous metabolic and cellular pathways, notably those controlling cell proliferation, differentiation, and survival [5, 6]. miRNAs function by binding with their target messenger ribonucleic acid (mRNA) to negatively regulate its expression. The complementarity between miRNA and the mRNA target determines how the gene will be silenced, either by cleavage of the mRNA or translation inhibition [7–9]. Overexpression of oncogenic miRNAs and loss of tumor suppressor miRNAs have been associated with tumorigenesis, progression, and metastasis in several cancers including HNC [10–12].

Recent studies have demonstrated that miRNAs are stably detectable in the blood and can serve as useful biomarkers for cancer [13–17]. Studies have reported differential miRNA expression in plasma/serum from patients with head and neck cancer compared to healthy controls [18–21]. As prognostic markers, miR-210, miR-21, and miR-375 have been associated with poor survival in laryngeal squamous cell carcinoma [22–24]. miRNA profiling assays followed by individual quantification could prove to be a robust method of screening hundreds of miRNAs from a variety of patient samples, although diversity in cut-off values, normalisation, and variability in sample size and sample collection still remain major challenges in the use of miRNAs as prognostic biomarkers in clinical practice.

A large number of head and neck cancer studies have investigated the expression level of circulating miRNAs in liquid biopsy samples, especially blood serum and plasma. Although previous narrative reviews and systematic reviews on meta-analysis studies have reported the prognostic significance of miRNAs in paired tissue samples from multiple types of head and neck cancers [25–27], there is still a lack of data on the prognostic role of blood-based miRNAs. Therefore, a pooled analysis of multiple miRNA expression profile studies for multiple types of head and neck cancer patients was performed to report the impact of miRNA levels on the patient's survival. Herein, we have systematically reviewed all articles investigating the prognostic value of high/low miRNA expression in various head and neck cancer patients. A meta-analysis was performed to further confirm the clinical relevance of previously reported miRNAs in selected head and neck cancer studies.

## 2. Materials and Methods

This meta-analysis was performed following the guidelines of the preferred reporting items for systematic reviews and meta-analysis (PRISMA) statement [28] and our previous work on the prognostic role of miRNAs in human non-small-cell lung cancer [80].

### 2.1. Search of Publications

We conducted a systematic literature search of Scopus, PubMed, ScienceDirect, Web of Science, and Medline between December 2006 and February 2019, using key words, including “microRNA expression” or “miRNA expression”; “Head and Neck Cancer”, “Head and Neck Squamous Cell Carcinoma”, or “HNSCC”; and “Prognosis”, “Human”, and “Overall Survival” (Supplementary [Supplementary-material supplementary-material-1]). We also performed a manual review of references from published articles to select additional studies that analysed associations between miRNA expression, head and neck cancer prognosis, and predictive impact.

### 2.2. Selection Criteria

PubMed search results were selected as it provided the most comprehensive literature search for the topic. The title and the abstract of all potentially relevant studies were evaluated for their contents ensuring adherence to the following inclusion and exclusion criteria for the meta-analysis.

Inclusion criteria are the following:

- Clinical studies that discussed prognostic value of high/low miRNAs related to only liquid biopsy samples in head and neck cancers
- Studies that published miRNA expression profile and clinicopathological and demographic data
- Studies exploring survival outcome and presenting associated hazard ratio (HR), 95% confidence interval (CI) for Disease-Free Survival (DFS), or Overall Survival (OS)

Exclusion criteria are the following:

- Studies without full text
- Studies not published in English
- Studies based on cancer tissues (paired tissues, formalin-fixed (FF), or formalin-fixed paraffin-embedded (FFPE))
- Studies representing a case control, letters, narrative reports, and reviews
- Molecular studies (cell line, *in vitro*/*in vivo* studies, and nonhuman)
- Studies not reporting miRNAs and their prognostic result

### 2.3. Data Extraction

Data were extracted independently from all the eligible studies for systematic review and meta-analysis. The retrieved data was prepared in a custom Microsoft Excel file for further evaluation of study quality and data synthesis. Prespecified data parameters included

- demographic data regarding population, ethnicity, and survival rates during follow-up
- tumor data (sample source, histology, stage, grade, and lymphoid node invasion)
- Experimental data involving study design, materials, assay technique, and dysregulation of selected miRNA expression
- Statistical data including hazard ratios for OS, 95% CI, and *P* value
- Publication data (author's name, publication year, and journal title)

### 2.4. Quality Assessment

Two reviewers critically assessed the quality of all the studies included in this systematic review and meta-analysis. The methodological quality was assessed by a quality assessment template based on the National Heart, Lung and Blood Institute (NHLBI) for systematic review and meta-analysis [29, 30]. All of the studies were categorised into three groups: “unsatisfactory,” “satisfactory,” and “good quality.” The cut-off score was designed as such that each study needed to be above “satisfactory” to be included for meta-analysis as described in Table 1.

### 2.5. Statistical Analysis

Comprehensive Meta-Analysis (CMA) software version 3.0 (Biostat, USA) was used to perform analysis of the accumulated data from selected studies. A forest plot was generated using head and neck cancer patient survival data (HR and 95% CI) to illustrate the association of miRNA expression and OS. Heterogeneity was assessed using the Cochran *Q* test and Higgins *I*-squared statistic where a *P* value less than 0.05 (*P* < 0.05) and *I*-squared value greater than 50% (*I*‐square > 50%) indicated the presence of significant heterogeneity across the studies [31]. The fixed or random effects model was applied to observe the heterogeneity between studies. An observed HR > 1 indicated a poor OS and poor prognosis in the group with elevated or reduced miRNA expressions. Publication bias was evaluated with the inverted funnel plot and Egger's and Begg's bias indicator test. All the *P* values were two-sided, with *P* value less than 0.05 (*P* < 0.05) considered statistically significant.

## 3. Results

### 3.1. Overview of the Included Studies

**Figure 1:**
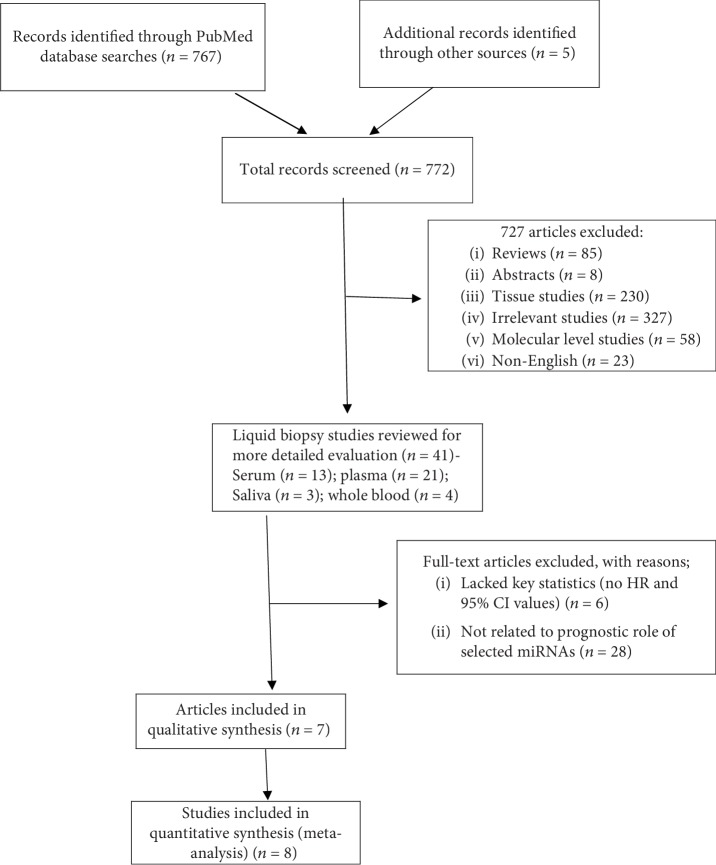
Flow chart of literature review and study selection process.

According to the described search criteria, a preliminary online PubMed search highlighted a total of 767 studies concerning dysregulation of miRNAs in multiple types of head and neck cancers. An additional manual search from references and citations of published articles was also performed and uncovered an additional five studies. A total of 727 records were excluded as they did not meet the initial inclusion criteria and represented irrelevant studies, molecular studies, literature reviews, and non-English and incomplete articles. As this study was focused on analysing liquid biopsy samples, 119 out of 230 cancer tissue (paired tissues, formalin-fixed (FF) or formalin-fixed paraffin-embedded (FFPE)) studies and those analysing miRNA expressions and their prognostic role were also excluded. All of the 41 studies relating to liquid biopsy samples including saliva, whole blood, blood serum, and blood plasma were included for further detailed evaluation. Out of the total 41 studies, only 13 studies [33–45] described the role of dysregulated miRNAs in head and neck cancer patient survival as Disease-Free Survival (DFS) and OS. An additional six articles were excluded as they lacked key statistics: HR and 95% CI values. Finally, a total of seven articles consisting of eight independent studies were included in the meta-analysis. One article by Wang and colleagues included two independent cohorts examining the role of miRNA signature in patient survival [43]. A flow chart of the study selection process is detailed in Figure 1. The scientific literature published from December 2006 to February 2019 was also interrogated separately in the PubMed search engine to analyse the role of circulating exosomal miRNAs in head and neck cancer prognosis. Out of the 36 search results, only one study reported circulating exosomal miRNA (miR-9) and provided statistical parameters (HR and 95% CI), hence included in our meta-analysis [38]. The flow chart for the selection of studies are presented in Supplementary [Supplementary-material supplementary-material-1]. All the studies reporting the circulating exosomal miRNAs in various head and neck cancers are also listed in Supplementary [Supplementary-material supplementary-material-1] [32, 47–53].

The complete clinical data from all seven studies included in the systematic review and meta-analysis are summarised in Table 2. There were a total of 1577 patients across all selected studies ranging from 18 to 256 patients. miRNA expression was analysed using a qRT-PCR assay for the blood samples, serum [37–40, 42, 45], and plasma [33–36, 41, 43, 44] obtained from multiple head and neck cancer types. Out of the 13 studies, 11 studies were conducted in China while two were conducted in Europe [33, 41]. All 13 studies reported the prognostic value of 22 different miRNAs explaining OS where 18 miRNAs were upregulated and four downregulated, impacting the patient outcome. Anatomic location and histological stage were explained clearly by all selected studies except one study by Qui et al. [40] which did not mention the tumor stage and grade.

### 3.2. Study Outcome

**Figure 2:**
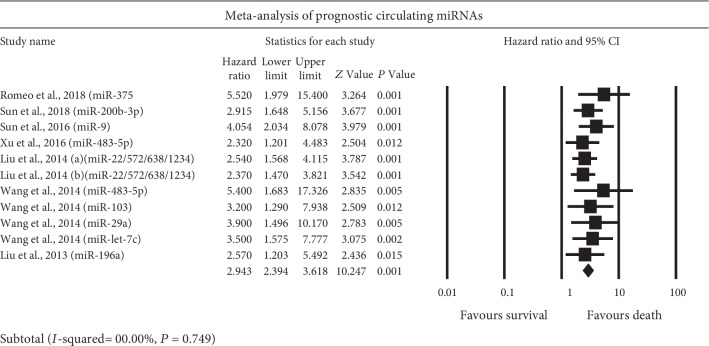
Forest plot for survival outcome of upregulated miRNAs in head and neck cancer patients.

All the selected studies for meta-analysis reported survival relationship of dysregulated miRNAs to predict HNC prognosis. Eighteen upregulated miRNAs (miR-375, miR-200b-3p, miR-24-3p, miR-483-5p, miR-29b, miR-191-5p, miR-374b-5p, miR-425-5p, miR-22, miR-572, miR-638, miR-1234, miR-103, miR-29a, mir-let-7c, miR-196a, miR-17, and miR-20a) and four downregulated miRNAs (miR-9, miR-187^∗^, miR-29c, and miR-223) were found associated with very poor survival in head and neck cancers. Only two miRNAs (miR-9 and miR-483-5p) were reported in more than one study; however, further subgroup analysis was not performed as they were reported in only two studies. The HRs and 95% CIs extracted from the studies were combined to interrogate the relationship between miRNA expression and head and neck cancer prognosis. The fixed effects model calculated the combined HR higher than 1.5 (HR > 1.5) suggesting very poor prognosis for multiple head and neck cancers. The combined HR (95% CI) for all eight studies was calculated as 2.94 (2.39-3.61; *P* < 0.001). No heterogeneity was observed among the selected studies (*I*‐squared = 00.00%, *P* = 0.749); hence, the fixed effects model was applied (Figure 2).

The studies included in this meta-analysis were all obtained from the currently available pool of multiple head and neck cancer studies, where the mean HR of any of the selected studies would fall within the range of cumulated confidence interval of hazard ratio. Among the eight independent cohorts, four studies were carried out on the blood plasma sample [33, 34, 43, 44] whereas the other four cohorts use blood serum samples for miRNA analysis [38, 39, 42]. The *Z* value (10.247, *P* < 0.001) calculated to test the null hypothesis, i.e., the risk of an event, was the same in both patient groups with high and low miRNA expressions, rejecting the null hypothesis concluding the higher risk of death within the group of patients representing significantly upregulated miRNAs.

### 3.3. Publication Bias and Sensitivity Analysis

**Figure 3:**
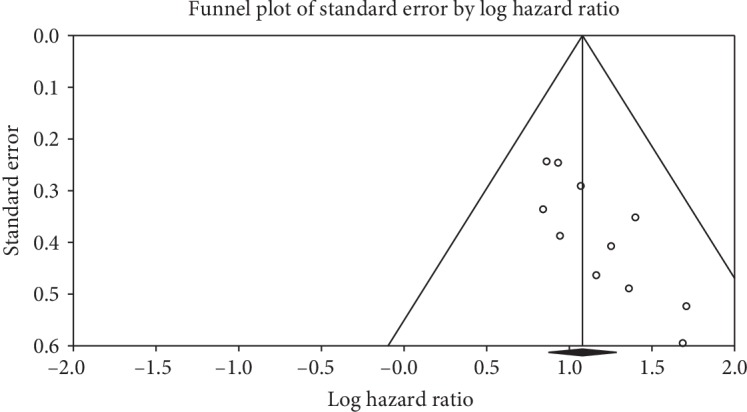
Funnel plot of studies included in meta-analysis correlating the patient survival and miRNA expression within multiple head and neck cancers.

Publication bias of the selected studies was interrogated by Begg's funnel plot and Egger's test (Figure 3). The two-tailed *P* value in Begg's test (*P* = 0.003) and Egger's test (*P* = 0.003) provided the statistical evidence of funnel plot asymmetry concluding apparent bias in the selected studies in meta-analysis.

To evaluate the sensitivity of each studies selected within the meta-analysis, we performed sequential omission of individual studies using a fixed effects model to calculate the pooled HR and CI. No study was found to influence the overall results.

## 4. Discussion

We conducted a comprehensive systematic review of the published literature on multiple head and neck-related cancers to establish a comprehensive picture of the circulating miRNA role in the patient survival. To the best of our knowledge, this is the first extensive report describing the prognostic role of circulating miRNAs in collective head and neck cancers, in which 13 studies involving 1577 patients were analysed and the relationship between miRNA expressions and prognosis was assessed. A total of 22 miRNAs involved in the survival analysis of head and neck cancers were compared. These findings showed that a significant elevated expression of eighteen miRNAs (miR-375, miR-200b-3p, miR-9, miR-24-3p, miR-483-5p, miR-29b, miR-191-5p, miR-374b-5p, miR-425-5p, miR-22, miR-572, miR-638, miR-1234, miR-103, miR-29a, mir-let-7c, miR-196a, miR-17, and miR-20a) were associated with poor survival in patients with head and neck cancer [33, 34, 37, 39, 40–45]. Moreover, the decreased expression of miR-9, miR-187^∗^, miR-29c, and miR-223 was also found associated with poor survival [35, 36, 38, 45, 46].

Two miRNAs (miR-9 and miR-483-5p) were identified by two studies each to be dysregulated in blood samples of head and neck cancer patients. In two of the studies [35, 38], miR-9 was found to be downregulated in blood samples when compared to healthy controls suggesting it as an independent risk factor for head and neck cancers. In both studies, the low miR-9 expression predicted poor overall survival rates. Previously, Lu et al. have suggested that miR-9 was underexpressed in oral squamous cell carcinoma (OSCC) tissues and cell lines by downregulating the expression of the CXC-chemokine receptor 4 via the Wnt/*β*-catenin signalling [54]. A study by Emmrich et al. also demonstrated that the reduced expression of miR-9 in nasopharyngeal carcinoma (NPC) tissues and cell lines was negatively correlated with clinical stage and metastasis [55]. Beyond head and neck cancers, miR-9 has been found to be downregulated in patients with acute myeloid leukemia [56] and gastric adenocarcinoma [57]. However, studies have also shown an oncogenic effect of miR-9 in various cancers. A study by Hu et al. has reported that upregulation of miR-9 in the serum of osteosarcoma patients was significantly associated with a poor overall survival duration, tumor stage, size, and metastasis [58]. Similarly, high levels of miR-9 in esophageal squamous cell carcinoma (ESCC) tumor tissue specimens were strongly associated with clinical progression, lymph node metastasis, and poor overall survival [59]. Zhu et al. reported significant upregulated miR-9 expression in glioma tissues associated with overall survival and tumor progression [60]. A study by Qu and colleagues also showed that overexpression of miR-9 in colorectal cancer (CRC) can enhance motility suggesting a crucial role in CRC metastasis [61]. These contradictory roles for miR-9 within various cancer types further suggest that miR-9 has the ability to act as both a tumor suppressor and an oncogene. It is also possible that the function of miR-9 may be cancer-dependent and cell-dependent as a single miRNA can target many downstream genes influenced by the tumor microenvironment [38].

Many studies have reported the association of miR-483-5p with patient's clinicopathological characteristics and prognosis in several cancer types such as multiple myeloma, NPC, and ESCC [62–66]. A miRNA profiling study by Xue et al. reported high expression levels of miR-483-5p associated with a poor outcome not only in plasma but also in cell lines and clinical tissues of NPC patients [63]. Similar association has been reported in ESCC tissues [64, 65]. Zheng et al. suggested a prognostic role in multiple myeloma patients as they reported an increased level of plasma miR-483-5p correlated with poor progression-free survival [62]. Similarly, Blot and colleagues reported significantly higher levels of miR-483-5p in postoperative serum of ESCC patients exhibiting lower survival rates than those with low levels of miRNA-483-5p [66]. Our current meta-analysis study also supports those previous findings that circulating miR-483-5p upregulated in patient sera might be used as an independent prognostic factor for predicting the clinical outcome of head and neck cancer patients [39, 43].

A previous meta-analysis by Sabarimurugan et al. has shown that significantly elevated miR-21 in head and neck squamous cell carcinoma (HNSCC) tissue samples was associated with poor survival outcomes [26]. A similar study by Jamali and colleagues has also demonstrated a similar association of upregulated miR-21 with HNSCC patient survival [25]. However, significant heterogeneity was observed in both studies, likely due to the differences in patient's clinicopathological characteristics (ethnicity, nationality, gender, age, tumor stage, and tumor grade), geographic location of the studies, assay methods, and cut-off criteria. The individual cohorts included in both meta-analysis studies analysed miRNA expressions through a variety of sample preparation methods (i.e., paraffin-fixed, formalin-fixed, freshly frozen tumors, or liquid biopsy samples), which could have highlighted heterogeneity likely due to data normalisation. In our current meta-analysis, the selected head and neck cancer studies utilised similar assay methods (i.e., qRT-PCR) to detect the circulating miRNAs from serum or plasma and applied similar normalisation procedure which might have resulted in no heterogeneity among the selected studies (*I*‐square = 00.00%). Moreover, the OS, consolidated HR, and 95% CI were statistically significant in all included studies indicating the poor prognostic role of any overexpressed or underexpressed miRNAs.

Previous studies have shown that tobacco use and alcohol consumption are one of the major risk factors for head and neck cancers [67, 68]. In fact, a study by Graham et al. has suggested an estimated 10-fold higher risk of developing HNSCC to cigarette smokers that can increases more with duration and extent of smoking [69]. Moreover, it is also suggested that alcohol consumption in conjunction with smoking can have synergistic effect that enhances the risk of developing HNSCC [70, 71]. Similarly, while exploring the role of tobacco use and alcohol consumption in overall head and neck cancer prognosis, only two studies selected in our current systematic review reported significant associations with patient's smoking status [35, 40] whereas only one study reported significant correlation of alcohol consumption on patient's survival [40]. Only one study included in our systematic review and meta-analysis explored the significant association of patient's age to cancer prognosis [35]. Sun and colleagues observed significant poor survival in male patients compared to female medullary thyroid cancer patients [34] whereas the rest of the studies did not report significant correlation of prognostic miRNAs with patient's sex.

This present study, despite focusing solely on blood-based biomarkers within head and neck cancer cases, shared a few interesting overlaps with previous studies observing miRNA expression and patient survival. Although miR-21 and miR-155 were considered as one of the most studied oncogenic miRNAs for head and neck cancer prognosis [24, 25, 72–74], the studies selected in our meta-analysis did not analyse them individually. This may be due to their low expression levels in sera compared to tissue samples or to lack of prognostic ability. Some reports show congruence between tissue and serum—for example, high expression of miR-29b was found to be significantly correlated with poor survival in both serum and tissue samples from oral cancer patients [40, 75]. However other studies have shown discordance between tissue and sera. Our study reported that miR-17, miR-20a, and miR-375 are upregulated in oral cancer blood sera predicting poor prognosis [33, 45]; however, the studies included in the previous meta-analysis studies in [24, 25, 76, 77] have reported all these three miRNAs significantly downregulated in OSCC tissues predicting poor outcomes. Similarly, upregulated plasma miR-375 was significantly associated with a poor survival in medullary thyroid cancer patients [33] but was found downregulated in tumor tissue from laryngeal squamous cell carcinoma (LSCC) patients predicting poorer prognosis [78]. Moreover, downregulated serum miR-29c in NPC predicted a worse outcome [45] but showed a positive association with DFS in paraffin-embedded NPC tissue specimens [79]. These contradicting outcomes suggest that the expression level of the same miRNAs may vary between the samples (tissue and liquid biopsy), and this variability and lack of reproducibility would need to be overcome for blood-based miRNA expression to be clinically useful and should be evaluated in a prospective dataset.

Some limitations must be considered when interpreting the results of this current study. First, the number of studies available was limited and did not include a wide category of liquid biopsy samples to represent inclusive picture of circulating miRNAs in head and neck cancers. Although no heterogeneity was observed within the selected studies, significant publication bias was observed which may be due to the selection of miRNAs without clear justification in some studies. Since the number of studies were lower, we could not perform further subgroup analysis to explain the individual miRNA role within multiple studies. The little overlap between studies, especially in terms of only few miRNA representations within selected studies, has raised the concern of more variability introduced in methods of sample preparation and miRNA extraction. As suggested by Higgins and Thompson [31], high intra-assay variation may have resulted high preanalytical and analytical variabilities although a similar assay method was applied to measure miRNA expression among selected studies. Moreover, the limited statistical parameters could have also led to the imprecise outcome as many prognostic miRNAs were not completely analysed and provided statistical parameters. A larger sample size may have improved the reliability of the conclusions individually as well as in our combined meta-analysis result.

Although the complete role of miRNAs is still under investigation, blood-based miRNAs are considered to be the novel tool for cancer detection and outcome prediction. Our previous meta-analysis study [80] has already demonstrated the prognostic role of liquid biopsy sample-related miRNAs in patient's overall survival in human non-small-cell lung cancer (NSCLC). A recent meta-analysis study of circulating miRNAs in glioma patients has also demonstrated that circulating miRNAs are capable of distinguishing glioma from healthy control [30]. Supporting these facts, our present study analysed 13 global studies relating to blood-based miRNAs as potential prognostic markers of head and neck cancer patients and has also identified a statistical association between several miRNA expressions and patient survival. However, biomarkers from biofluid need to be better evaluated in order to improve minimally invasive and rapid approach for cancer detection and therapy monitoring in head and neck cancers.

## 5. Conclusion

To our knowledge, this is the first comprehensive meta-analysis that examines the prognostic roles of blood-based miRNAs separately in head and neck cancer patient's survival. Several miRNAs are established to play critical roles in the overall survival outcome by functioning either as oncogenes or as tumor suppressors. Levels of 22 miRNAs including miR-9 and miR-483-5p were found consistently up- and downregulated in multiple head and neck cancers and were associated with significantly poor survival. Validation of these blood-based miRNAs in large-scale standardized protocol-based prospective clinical studies is required before these can be considered for routine clinical use as diagnostic and prognostic biomarkers.

## Figures and Tables

**Table 1:** Quality assessment of the selected studies for meta-analysis.

S. no.	Criteria	Unsatisfactory (0-33%)	Satisfactory (34-66%)	Good (67-100%)
1	The objective of this study stated	—	—	13
2	Eligibility criteria	6	-3	4
3	Sample size justification	—	—	13
4	Target population	—	—	13
5	Cut-off criteria (follow-up)	—	1	12
6	Definition of anatomical site	—	—	13
8	Definition of the assay used	—	—	13
9	Outcome assessment (OS, DFS)	—	3	10
10	Outcome measures (HR, CI)	6	—	7
	*Total selected studies*	**6**	**3**	**4**

**Table 2:** Overview characteristics of the selected study in systematic review and meta-analysis.

S. no.	Study	Population	Study period	Sample number (patient/control)	Source of miRNA	miRNA/dysregulation	Cancer site	Histological stage	Tumor grade	Lymph node metastasis	miRNA analysis platform	Follow-up
1	Romeo et al. [33]	Italy	2011-2015	26/19	Plasma	miR-375/upregulated	Medullary thyroid	T1/T2 = 5; T3/T4 = 13; U = 1	N/A	N0 = 1; N1a/b = 17; U = 1	qRT-PCR	80 months

2	Sun et al. [34]	China	June 2004-June 2014	80/80	Plasma	miR-200b-3p/upregulated	Oral cavity	T1/T2 = 43; T2/T3 = 37	I = 48; II‐III = 32	N/A	qRT-PCR	>5 years

3	Lu et al. [35]	China	2007-2015	110/60	Plasma	miR-9/downregulated	Nasopharynx	T1 = 27; T2 = 24; T3 = 28; T4 = 31	I = 7; II = 19; III = 39	M0 = 106; M1 = 4	qRT-PCR	60 months

4	Liu et al. [36]	Taiwan	N/A	63/26	Plasma	miR-187^∗^/downregulated	Buccal mucosa = 35; tongue = 15; others = 13	T1‐T3 = 43; T4 = 20	I‐III = 35; IV = 28	N0 = 42; N > 0 = 21	qRT-PCR	46.2 months

5	Ye et al. [37]	China	2011-2014	65/20	Serum	miR-24-3p/upregulated	Nasopharynx	T1/T2 = 15; T3/T4 = 50	I‐II = 11; III‐IV = 54	N0‐N1 = 10; N2‐N3 = 55	qRT-PCR	50 months

6	Sun et al. [38]	China	N/A	104/40	Serum	miR-9/downregulated	Tongue = 34; nontongue = 70	T1‐T2 = 66; T3‐T4 = 38	I‐II = 59; III‐IV = 45	NNM = 63; N = 41	qRT-PCR	5 years

7	Xu et al. [39]	China	June 2008-September 2010	101/103	Serum	miR-483-5p/upregulated	Oral cavity	T1/T2 = 51; T3/T4 = 50	N/A	N/A	qRT-PCR	52.16 months

8	Qui et al. [40]	China	July 2011-August 2012	193/65	Serum	miR-29b/upregulated	Nasopharynx	N/A	N/A	M193	qRT-PCR	5 years

9	Summerer et al. [41]	Germany	N/A	18/12	Plasma	miR-191-5p/upregulated; mir-374b-5p/upregulated; miR-425-5p/upregulated	Larynx = 5; oropharynx = 6; oral cavity = 3; maxillary sinus = 1; paranasal sinuses = 1; nasopharynx = 1; hypopharynx = 1	T1 = 4; T2 = 2; T3 = 6; T4 = 6	N/A	N0 = 4; N1 = 4; N2 = 10 M0 = 16; M1 = 2	qRT-PCR	55.1 weeks

10	Liu et al. [42]	China	January 2001-December 2006	512	Serum	miR-22/upregulated; miR-572/upregulated; miR-638/upregulated; miR-1234/upregulated	Nasopharynx	T1 = 4; T2 = 31; T3 = 47; T4 = 46	N/A	M0 = 512	Microarray/qRT-PCR	5 years

11	Wang et al. [41]	China	January 2009-April 2009	50/50	Plasma	miR-483-5p/upregulated; miR-103/upregulated; miR-29a/upregulated; miR-*let*-7c/upregulated	Nasopharynx	T1‐T2 = 22; T3‐T4 = 78	N/A	N/A	qRT-PCR	77 months

12	Liu et al. [44]	Taiwan	N/A	95/24	Plasma	miR-196a/upregulated	Buccal mucosa = 34; tongue = 25; others = 36	T1‐T3 = 26; T4 = 59	I‐III = 26; IV = 69	N0 = 50; N > 0 = 45	qRT-PCR	32 months

13	Zeng et al. [45]	China	N/A	160/143	Serum	miR-17/upregulated; miR-20a/upregulated; miR-29c/downregulated; miR-223/downregulated	Nasopharynx	T1 = 2; T2 = 25; T3 = 55; T4 = 64	N/A	N/A	qRT-PCR	35 months

miRNA/miR: microribonucleic acid; qRT-PCR: quantitative real-time polymerase chain reaction; U: unknown; N: nodal; M: metastasis; N/A: not available; NNM: no nodal metastasis.

## Supplementary Materials

Supplementary Materials The scientific literature published from December 2006 to February 2019 was interrogated using 5 different search engines Scopus, PubMed, Science Direct, Web of Science, and Medline using key search words including “microRNA expression or miRNA expression”, “Head and Neck Cancer”, “Prognosis”, “Human”, and “Overall Survival”. Literature search results are noted in Supplementary Table 1. Supplementary Table 1: literature search result for systematic review and meta-analysis. The scientific literature published from December 2006 to February 2019 was also interrogated in PubMed search engine to analyse the role of circulating exosomal miRNAs in head and neck cancer prognosis using key words including “Exosomal microRNA expression”, “Head and Neck Cancer”, and “Prognosis”. Out of the 36 search results, only one study reported circulating exosomal miRNA (miR-9) and provided statistical parameters (hazard ratio and 95% confidence interval), hence included in our meta-analysis [38]. The flow chart for the selection of studies is presented in Supplementary Figure 1. All the studies that reported the circulating exosomal miRNAs in head and neck cancers are listed in Supplementary Table 2. Supplementary Figure 1: flow chart of literature review and study selection process for circulating exosomal miRNAs. Supplementary Table 2: characteristics of circulating exosomal miRNA studies interrogated during literature review.
