# Supplementary material for: Circulating MicroRNAs as Prognostic Molecular Biomarkers in Human Head and Neck Cancer: A Systematic Review and Meta-Analysis
Source: Dis Markers. 2019 Nov 18;2019:8632018. doi: 10.1155/2019/8632018 (PMC6885815; doi:10.1155/2019/8632018)
Supplement: Supplementary Materials — The scientific literature published from December 2006 to February 2019 was interrogated using 5 different search engines Scopus, PubMed, Science Direct, Web of Science, and Medline using key search words including “microRNA expression or miRNA expression”, “Head and Neck Cancer”, “Prognosis”, “Human”, and “Overall Survival”. Literature search results are noted in Supplementary Table 1. Supplementary Table 1: literature search result for systematic review and meta-analysis. The scientific literature published from December 2006 to February 2019 was also interrogated in PubMed search engine to analyse the role of circulating exosomal miRNAs in head and neck cancer prognosis using key words including “Exosomal microRNA expression”, “Head and Neck Cancer”, and “Prognosis”. Out of the 36 search results, only one study reported circulating exosomal miRNA (miR-9) and provided statistical parameters (hazard ratio and 95% confidence interval), hence included in our meta-analysis [38]. The flow chart for the selection of studies is presented in Supplementary Figure 1. All the studies that reported the circulating exosomal miRNAs in head and neck cancers are listed in Supplementary Table 2. Supplementary Figure 1: flow chart of literature review and study selection process for circulating exosomal miRNAs. Supplementary Table 2: characteristics of circulating exosomal miRNA studies interrogated during literature review. [file 8632018.f1.pdf]

Supporting Information

The scientific literature published from December 2006 to February 2019 was interrogated using 5 different search engines; Scopus, PubMed, Science Direct, Web of Science and Medline using key search words including “microRNA expression or miRNA expression’, ‘Head and Neck Cancer’, ‘Prognosis’, ‘Human’ and ‘Overall Survival’. Literature search results are noted in **supplementary table 1**.

Supplementary table 1: Literature search result for systematic review and meta-analysis.

S.N	Keyword	Scopus	PubMed	Science Direct	Web of Science	Medline
1	Head and Neck Cancer	46,484	311,316	45,968	23,522	838
2	miRNA expression	8,852	60,680	12,467	8,534	37
3	Prognosis	876,089	1,625,231	574,795	325,739	12,126
4	Head and neck cancer AND miRNA expression	52	2,149	348	37	7
5	Head and neck cancer AND prognosis	7,333	69,318	14,129	1,965	175
6	miRNA expression AND prognosis	1,327	7,778	3,746	1,031	12
7	Head and neck cancer AND miRNA expression AND prognosis	22	767	246	10	7

The scientific literature published from December 2006 to February 2019 were also interrogated in Pubmed search engine to analyse the role of circulating exosomal miRNAs in head and neck cancer prognosis using key words including " Exosomal microRNA expression', 'Head and Neck Cancer', 'Prognosis'. Out of the 36 search results, only one study reported circulating exosomal miRNA (miR-9) and provided statistical parameters (Hazard Ratio and 95% Confidence Interval), hence included in our meta-analysis [38]. The flow chart for the selection of studies are presented in **supplementary figure 1**. All the studies those reported the circulating exosomal miRNAs in head and neck cancers are listed in **supplementary table 2**.

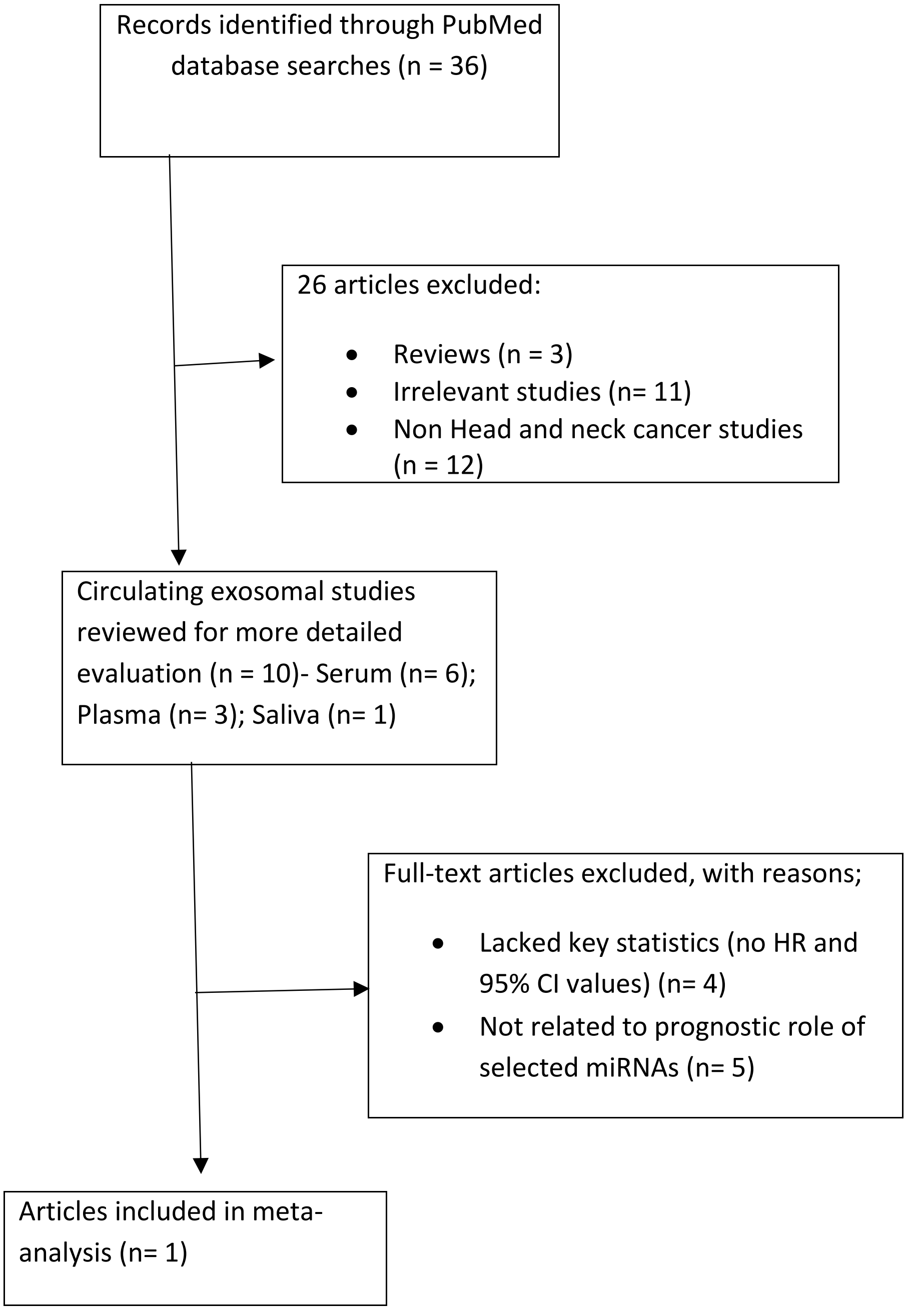

Supplementary figure 1: Flow chart of literature review and study selection process for circulating exosomal miRNAs.

Supplementary table 2: Characteristics of circulating exosomal miRNA studies interrogated during literature review.

S. No.	Study	miRNA/Dysregulation	Source of exosomal miRNAs	Role of miRNA
1	Qin et al, 2019 [32]	miR-196a/Upregulated	Plasma (Head and Neck Cancer)	Prognosis (Only P value)
2	Tomasetti et al, 2018 [47]	miR-126/downregulated	Serum (Intestinal-type Sinonasal Adenocarcinoma)	Tumor suppression
3	Lu et al, 2018 [35]	miR-9/Downregulated	Plasma (Nasppharyngeal Carcinoma)	Prognosis (Included in the Meta-Analysis)
4	Bao et al., 2018 [48]	miR-23a/Upregulated	Serum (Nasopharyngeal Carcinoma)	Angiogenesis/prognosis (Only P value)
5	Langevin et al, 2017 [49]	miR-486-5p/miR-486-3p/Upregulated	Saliva (Head and Neck Squamous Cell Carcinoma)	Diagnosis
6	Samsonov et al, 2016 [50]	miR-21/upregulated miR-181a-5p/Upregulated	Plasma (Papillary Thyroid Cancer)	Diagnosis
7	Ye et al, 2016 [37]	miR-24-3p/Upregulated	Serum (Nasopharyngeal Carcinoma)	Prognosis (Only P value)
8	Li et al, 2016 [51]	miR-21/Upregulated	Serum (Oral Squamous Cell Carcinoma)	Cell migration and Invasion
9	Wang et al, 2014 [52]	miR-21/Upregulated	Serum (Laryngeal Squamous Cell Carcinoma)	Diagnosis
10	Huang et al, 2018 [53]	miR-1246, miR-1290, miR-335-5p, miR-127-3p, miR-122-5p/Upregulated	Serum (Laryngeal squamous cell Carcinoma)	Progression
